# Biotin-Streptavidin Binding Interactions of Dielectric Filled Silicon Bulk Acoustic Resonators for Smart Label-Free Biochemical Sensor Applications

**DOI:** 10.3390/s140304585

**Published:** 2014-03-07

**Authors:** Amir Heidari, Yong-Jin Yoon, Woo-Tae Park, Pei-Chen Su, Jianmin Miao, Julius Tsai Ming Lin, Mi Kyoung Park

**Affiliations:** 1 Institute of Microelectronics, Agency for Science, Technology and Research (A * STAR), Singapore 117685, Singapore; E-Mails: aheidari@ucdavis.edu (A.H.); tsaiml@ime.a-star.edu.sg (J.T.M.L.); parkmk@ime.a-star.edu.sg (M.K.P.); 2 Department of Mechanical and Aerospace Engineering, Nanyang Technological University, Singapore 639798, Singapore; E-Mails: peichensu@ntu.edu.sg (P.-C.S.); jmiao@pmail.ntu.edu.sg (J.M.); 3 Department of Mechanical and Automotive Engineering, Seoul National University of Science and Technology, Seoul 139743, Korea; E-Mail: wtpark@seoultech.ac.kr; 4 InvenSense Inc., San Jose, CA 95110,USA

**Keywords:** biosensor, capacitive filled gap transducer, biotin, streptavidin

## Abstract

Sensor performance of a dielectric filled silicon bulk acoustic resonator type label-free biosensor is verified with biotin-streptavidin binding interactions as a model system. The mass sensor is a micromachined silicon square plate with a dielectric filled capacitive excitation mechanism. The resonance frequency of the biotin modified resonator decreased 315 ppm when exposed to streptavidin solution for 15 min with a concentration of 10^−7^ M, corresponding to an added mass of 3.43 ng on the resonator surface. An additional control is added by exposing a bovine serum albumin (BSA)-covered device to streptavidin in the absence of the attached biotin. No resonance frequency shift was observed in the control experiment, which confirms the specificity of the detection. The sensor-to-sensor variability is also measured to be 4.3%. Consequently, the developed sensor can be used to observe in biotin-streptavidin interaction without the use of labelling or molecular tags. In addition, biosensor can be used in a variety of different immunoassay tests.

## 1. Introduction

Although the chemical/biochemical sensor market growth rate is relatively high, the majority of chemical analyses are still dependent on costly bench-top instruments laboratory analyses. They are highly sensitive and selective, but the analyses are performed off-line and typically are time and labor intensive, thus limiting their applications. Therefore, inexpensive, highly sensitive chemical/biochemical sensors with ability of quick measurement are still in great demand [1,2].

From this point of view, micromachined chemical/biochemical sensors, which make use of well-established semiconductor fabrication processes, are one of the most promising candidates [2,3]. They provide an interesting means to analyze the content of a biological sample based on the direct conversion of a biological event to an electronic signal and allow highly sensitive and selective detection of micro-organisms or toxins. Compared to other competing technologies, low power consumption, small sensor and sample volumes (e.g., blood), fast response times, excellent manufacturing/process repeatability and mass production capability with high production yield are strong advantages of micromachined technology.

Other types of micromachined biosensors like surface vibration spectroscopy [4], optical sensors [4,5], quartz crystal microbalances (QCMs) [6,7], film bulk acoustic resonators (FBARs) [8,9] and microcantilevers [10,11] have been used for label-free study of biomolecular interactions. Among these, QCMs have been extensively developed for label-free monitoring of affinity interactions between molecules with real-time output, high sensitivity and good specificity [12,13]. Several groups have previously demonstrated micro/nanomechanical resonant mass sensors based on flexural mode resonators [14,15]. Flexural mode resonators have limited quality factors in liquid, thereby limiting the achievable mass resolution. Further miniaturization using nanofabrication techniques allows higher mass sensitivity, but it includes the low device-to-device performance reproducibility and difficulties to electrically interface to measurement equipments [16]. The designed biomass sensor in this work is a micromachined silicon square plate with a capacitive excitation mechanism which shows a good potential to detect small biological agents at aqueous environment. The capacitive gap of the transducer is filled with silicon nitride which helps to achieve smaller gap distance in comparison with air gap counterparts [17]. Filling the gap also provides some other benefits since it better stabilizes the resonator structure against shocks and eliminates the possibility of particles or liquid getting into the electrode-to-resonator air gap, which poses a potential reliability issue.

To characterize the fabricated resonator, two different methods were used. In the first method, layer-by-layer self-assembly polymer known as polyelectrolyte multilayer (PEM) was used to coat the resonator surface [18,19]. After coating a specified number of layers of the PEM (a specific amount of mass), the resonance frequency shift was measured. Since, PEM was formed by the sequential deposition of two oppositely charged polyelectrolytes, the frequency shift could be measured after each layer deposition. In the second method, the well characterized biotin-streptavidin interaction was used as a model system. The interaction between biotin-streptavidin is one of the highest non-covalent affinities known with a K_d_ ≈ 10^−14^–10^−16^ M [20,21] and was used for characterization of micro- and nanomechanical biosensors [3,22,23].

## 2. Experiment

### 2.1. Design and Fabrication of the Lamé Mode Sensor

**Figure 1.**
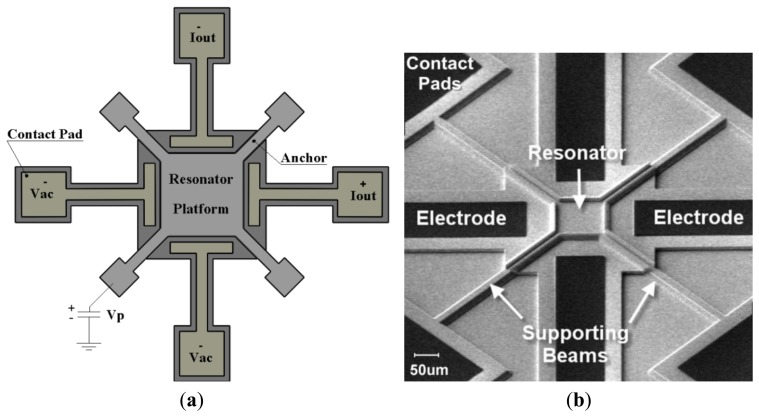
(**a**) Schematic of the device showing the resonator, exciting and sensing electrodes, driving and biasing setup (**b**) SEM microgrpagh of the fabricated mass sensor, silicon resonator, support beams and its surrounding polysilicon electrodes.

The details of the design procedure and characterization of the sensor can be found in [24,25]. The sensor structure consists of a thin square resonator supported by four anchors at the corners and surrounded by four drive/sense electrodes (Figure 1a). The square plate is separated from the surrounding electrodes with a 80 nm nitride gap that defines the capacitance of the electro-mechanical transducer. The resonator is laterally driven with electrodes on two opposite sides of the square plate symmetrically. A DC-bias voltage (*Vp*) is applied to the structure via the anchors, while two AC input voltages are applied to the input electrodes with 180° of phase difference. These voltages result in a time-varying electrostatic force on the plate edges which makes it oscillate in its fundamental frequencies in the Lamé mode. The capacitive gap distances of the opposite side change with this same frequency. Because of the electrostatic field in the gap, a time-varying current is induced in the output electrode. A SEM micrograph of the square resonator, showing the resonator and its surrounding electrodes is depicted in Figure 1b.

If the material of the square plate is homogeneous and isotropic, and if the side length of the square, *L* is much larger than its thickness, the square resonator can be theoretically modelled as a thin plate. In this case, the resonant frequency of Lamé mode can be calculated as below [18]:

(1) $${\text{f}}_{0}=\frac{\text{n}}{\sqrt{2}\text{L}}\sqrt{\frac{{\text{C}}_{44}}{\rho }}$$

where *n* is the order of the resonance mode, *ρ* is the material density and *C_44_* is stiffness constant which is the shear modulus of the silicon *G*. For the single-crystal silicon structure, the material properties are as follows: *ρ* = 2330 kg/m^3^ and *G* = 70 GPa. Replacing these values in Equation (1), the corresponding resonant frequency of a square resonator with *L* = 100 *μm* will be *f*_0_ = 38.75 MHz.

Mass sensitivity of the biosensor is defined as the shift in the sensor resonance frequency due to the changes in the mass. A higher mass sensitivity helps to measure smaller masses. The mass sensitivity *S_m_* of the bulk-wave resonator mass sensor was defined by Sauerbrey as below [26]:

(2) $$S_{m}=\frac{1}{2\text{m}}=\frac{1}{2\rho_{r}\text{t}}$$

The definition of the mass sensitivity depends on the density of the resonator structure material (*ρ_r_*) and resonator's thickness (*t*) and is valid for the sensor vibrating in the air. The mass sensitivity of the sensor (*S_m_*) in the air for the fabricated devices was analytically evaluated to be 107.29 μm^2^/ng for a 38.75 MHz resonator. Assuming a mass load of a Newtonian liquid with a mass of *Δm* = *Aρδ*, Equation (2) results in the well-known Kanazawa and Gordon [27], who related the product of the density and viscosity of the liquid to the frequency shift of the resonator:

(3) $$\Delta f=-f_{0}^{3/2}\sqrt{\frac{\eta_{L}\rho_{L}}{\pi \rho_{r}{\bar{C}}_{66}}}$$

where *ρ_L_* and *η_L_* are the density and viscosity of Newtonian liquid and *C̄*_66_ is the element of tensor elasticity which can be replaced with Young's modulus (*E*) here. An analytical expression for the mass sensitivity of the biosensor in liquid environment can be obtained using the same procedures described in [28]. The expression for the mass sensitivity of a laterally vibrating beam is then the same as the expression for the in-plane square resonator. If the thickness of the sensing layer is considered small enough, then the mass sensitivity can be approximated as:

(4) $$S_{m,\mathrm{approx}}=\left|\frac{\alpha_{i}^{2}}{2\pi }\frac{\sqrt{\mathrm{Et}}}{4\sqrt{3}{\left(M_{\mathrm{eff}}\right)}^{3/2}}\right|$$

where *α_i_* is a constant dependent on the mode number (*α_i_* = 1.875 for the fundamental in-plane mode), *E* is the Young's modulus and *t* is the resonator thickness. When the beam is operating in air or low viscosity media, the effective mass can be approximated as the beam mass, *M_eff_* = *ρ_B_L*^2^*t*, where *ρ_B_* denotes the beam density, *L* is the square resonator length. Since the chemical sensitivity is proportional to the mass sensitivity multiplied by the volume of the sensing layer (e.g., *L*^2^*t*_2_, where *t*_2_ is the thickness of the sensing layer) the chemical sensitivity is predicted to be proportional to (*t*_2_/*t*)*(b/*L*^2^). As the resonant frequency is known to be proportional to *b*/*L*^2^ e.g., [29]), beams with higher resonant frequencies are then predicted to have higher chemical sensitivities.

The resolution of biosensors is limited by Limit of Detection (LOD). LOD describes the smallest amount of mass that a sensor can detect considering the existing noise. The uncertainty in the measured frequencies can be caused by device imperfection, the measurement device, and environment noise. The LOD of an acoustic mass sensor is given by [30]:

(5) $$\mathrm{LOD}=\frac{N}{S_{m}}$$

where *N* is the noise level of the sensor, equal to 3 times the standard deviation of measured resonance frequency of a bare resonator (*N* = 3σ)The noise and LOD are calculated for the developed resonators in following sections.

### 2.2. Sensor Surface Preparation

**Figure 2.**
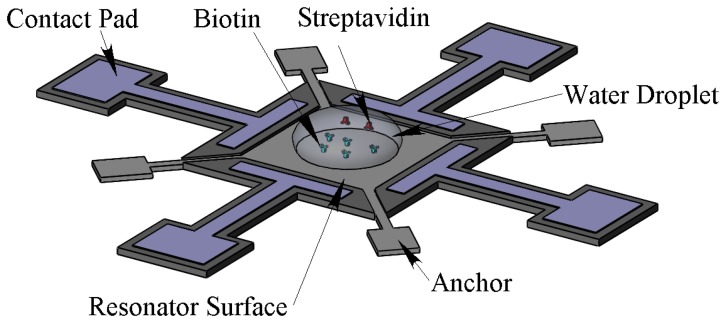
Schematic of the resonator and water droplet containing streptavidin spotted on the center of biotin modified resonator far from the surrounding electrodes. The maximum water droplet volume that can be applied to each resonator is assumed to have covered 78% of the top surface from optical inspection.

In the remainder of this paper, the mass sensitivity of the developed sensor is experimentally demonstrated in both air and aqueous environments. For this purpose, PEM was used as a controllable mass on the resonator surface. Then the mass sensitivity was estimated with biotin-streptavidin in the aqueous environment. In order to test in the aqueous environment, the resonator surface is spotted with a water droplet. As the viscous damping increases when the droplet contacts the surface, both frequency and Q-factor will be affected. The maximum droplet volume that can be applied is assumed to be the droplet volume that has a water contact area of approximately πL^2^/4 (*i.e.*, 78% of the top surface area) which is π*L*^3^/12. For the resonator with side length of *L* = 100 μm, the droplet volume is approximately 0.3 nL. The water droplets are spotted on the centre of the resonators using a KRÜSS-DSA100 contact angle measurement instrument. As the minimum droplet size of this machine is 1.5 μL, to get the right volume, time has to pass to let the water evaporate. The evaporation time for a 1.5 μL water droplet to dry out completely is more than 30 min. As a result, the desired droplet in the resonator centre can be obtained before this time. Figure 2 shows a schematic of the resonator and water droplet containing streptavidin spotted on the center of biotin modified resonator far from the surrounding electrodes.

The frequency response and Q-factor of the resonators were measured after spotting the water droplet. The resonant frequency and extracted quality factor for the measured data are 32.42 MHz and 240, respectively. As expected the Q-factor of the resonator extremely decreased after spotting the water droplet on its surface as viscous damping in water is much higher than what is in the air.

### 2.3. Materials and Reagents

3-Aminopropyltriethoxysilane (APTES), poly(sodium-4-styrenesulfonate) (PSS), poly(allylamine hydrochloride) (PAH), and bovine serum albumin (BSA) were purchased from Sigma-Aldrich (St. Louis, MO, USA). Immunopure streptavidin and EZ-Link NHS-PE4-biotin were purchased from Thermo Scientific Pierce (Singapore). Other chemicals were analytical reagent grade and were used as received. All samples and buffers were prepared using deionized (DI) water obtained from a Milli-Q water purification system.

### 2.4. Polyelectrolyte Multilayer (PEM) Coating

The detailed procedure for PEM coating has been previously described [23]. In brief, the silicon resonator was first treated with a solution of 2% APTES in a mixture of ethanol/H_2_O (95%/5%, *ν*/*ν*) for 2 h, followed by thorough rinsing with ethanol and DI water. As the APTES make a positive charged layer on silicon surface, the coating process starts with the negatively charged polymer (PSS) and followed by the positive charged polymer (PAH). Polymer films were built by alternately dipping the chip in aqueous solutions of PAH (1 mg/mL) and PSS (1 mg/mL) for 15 min each. After each polymer adsorption, the chip was rinsed two times in deionized (DI) water with the assist of shaker table for 5 min. PEM was formed by successive dipping in aqueous PSS and PAH solutions.

### 2.5. Biotin-Streptavidin Immobilization

Four identical resonators (with the length of *L* = 100 μm) were prepared. The resonator surfaces were firstly cleaned using oxygen plasma for 60 s. This step also promotes the availability of surface hydroxyls for reaction with the silane group in the subsequent step. In the next step, the silicon resonator surfaces were modified with a 2% solution of 3-aminopropyltriethoxysilane (APTES) for 2 h similar to the process done in the previous section. After silanization, unbound silane was removed by three washes with ethanol. Three APTES-treated chips were functionalized with biotin (1 mg/mL) by incubating the resonators in solution for 1 h. One of the resonators was selected as the reference and its surface was blocked with a 100 μg/mL solution of BSA. In order to measure the resonator frequency shifts, the resonators were first dried with nitrogen gas. Then, to measure the resonance frequency in the aqueous environment, a water droplet was spotted on the resonator top surface as described in Section 2.3. The measured resonance frequency of each resonator was considered as the reference frequencies (f_0_) for that specific resonator.

**Figure 3.**
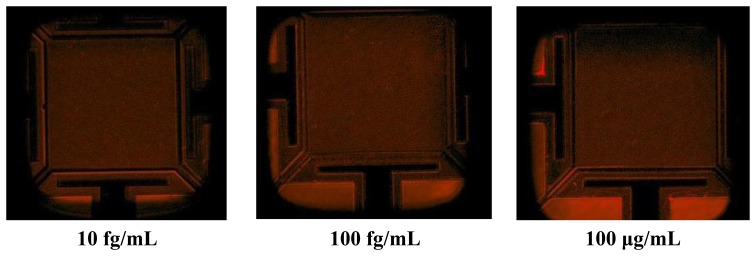
Flourecent micoscopy images of the resonators after streptavidin immobilization. The resonators are coated with different streptavidin concentrations and imaged separately using a same combination of excitation and emission filters.

The next step was to expose the streptavidin with different concentrations on the biotin modified resonator. Before starting the frequency shift measurement, the biotin–streptavidin reaction was confirmed through fluorescent microscopy with streptavidin molecules labelled with fluorescent dye, DyLight 633 (Thermo Scientific, Rockford, IL, USA) (Figure 3).

The fluorescent microscopy confirmed that 5 min after the introduction of the streptavidin solution, the interaction occurred and a strong fluorescent signal was obtained. In fact, the biotin–streptavidin binding only needs a few seconds to occur because of high affinity interaction. Then, sensors were exposed to to streptavidin solutions with concentration in the range of 1.88 × 10^−15^ M (100 fg/mL) to 1.88 × 10^−5^ M (1 mg/mL). The chip was only kept for 15 min in streptavidin solution to bind with biotin for each concentration.

## 3. Result and Discussion

### 3.1. Frequency Measurement and Mass Sensitivity Estimation Using PEM

**Figure 4.**
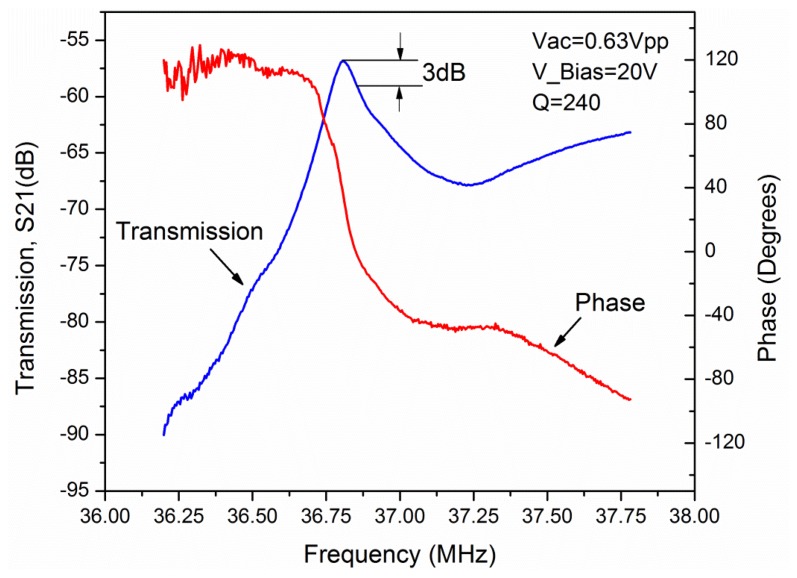
Measured S21 transmission of 36.8 MHz square resonator under DI water droplet (top surface contacted with water).

The chips were attached to a custom made printed circuit board (PCB) and the measurements were performed with an Agilent 4395A network analyser (Agilent, Santa Clara, CA, USA). This network analyzer was used to apply the AC voltage to the sensor and measure the output signal. The initial measurements were performed for bare resonators under water droplet, to get the resonance frequency and the Q-factor (Figure 4). S21 parameter is measured for an L = 100 μm resonator. The resonance frequency of this resonator was measured at 36.8 MHz with a Q value of 240 in liquid environment. The resonator was biased with a DC voltage of 20 V and an AC drive voltage of 0.63 Vpp.

The difference between the theoretical frequency (38.75 MHz) and the measured frequency (36.8 MHz) can be related to the difference in the material properties like Young's modulus used in the simulation (E = 169 GPa) and as its real value.

**Figure 5.**
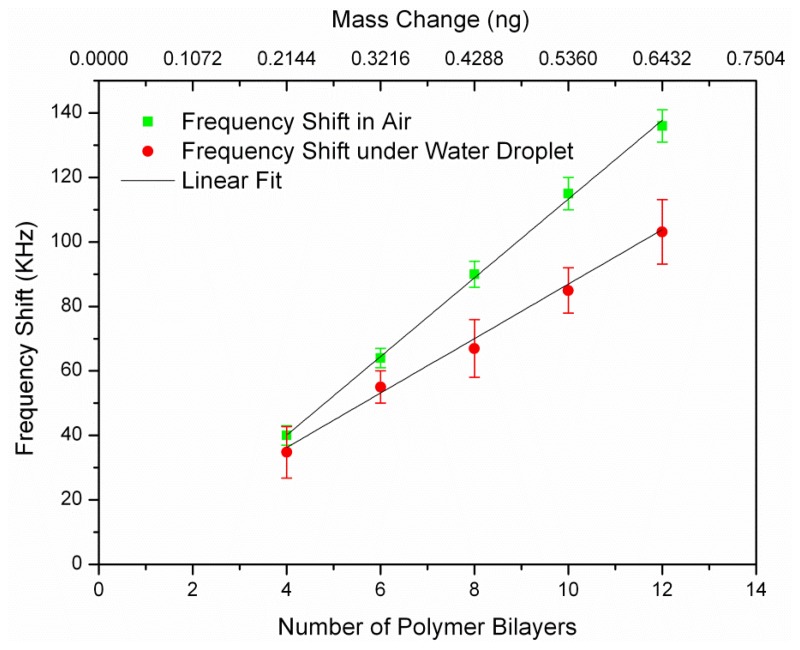
Absolute frequency shift, |*Δf*_(_*_i_*_)_| *versus* number ofcoated PEMbilayers and its corresponding mass change for the *L* = 100 μm resonator.

Before coating, the resonance frequency of non-coated resonators (after functionalizing their surface with APTES) was measured and considered as the reference frequency (*f*_0_) for all later frequency shift calculation (*Δf*_(_*_i_*_)_ = *f*_(_*_i_*_)_ − *f*_0_). The notation *f*_(_*_i_*_)_ refers to the resonance frequency of the sensor after coating of *i*-th polymer bilayer (*i* = 4,6, …,12). The chips were stored in nitrogen box for a few hours to be dried after each polymer coating step. The polyelectrolyte coating was known to be not uniform up to four bilayers [7]. As a result, the frequency shift would not be linear below four bilayers and thus we measured the first frequency shift after coating of four bilayers. Thin polymer film successfully formed by the successive deposition of PAH and PSS up to 12 bilayers on the resonators surface. Five frequencies values were recorded for each resonator after every 2-bilayer coatings to calculate the error bar. Figure 5 shows the measured frequency shifts (*Δf*_(_*_i_*_)_) in air and water for a *L* = 100 μm resonator *versus* the number of polymer coated bilayers and their corresponding mass. It can be seen that the frequency shift was linearly correlated with the number of coated polymer bilayers for all of the resonators.

We measured the mass sensitivity values of the resonator for two different conditions: (1) Bulk acoustic resonator in air, which corresponded to minimum damping condition; (2) Bulk acoustic resonator immersed in water, which was achieved by exposing the top surface of the resonator to a macro size water droplet which presented a semi-infinite thickness water layer. We characterized the mass sensitivity values of the resonators by measuring the two-port reflection coefficient (*S*_21_) using a network analyzer (Agilent 4395A). The mass sensitivity of the resonator could be estimated using the Equation (2) for air, measured frequency shifts from Equations (3) and (4) for liquid environment. Frequency shift in the liquid environment had larger error bar comparing to air as it is more difficult to read the exact resonant frequency in the lower Q-factor. The obtained mass sensitivity from both air and water were calculated and summarized in Table 1. The average *S_m_* in the air is 105.4 μm^2^, which is too close to the theoretical mass sensitivity (107.29 μm^2^/ng). The average calculated mass sensitivity of the resonator with the water droplet is found to be 79.53 μm^2^/ng.

For a high *Q* resonance, the acoustic energy in the resonator structure had to be well trapped. But when one side or both sides of the resonator was contacted liquid, the longitudinal acoustic-wave energy was no longer well confined in its resonating film. Some of it leaked into the contacting liquid, and was lost there since liquid drastically attenuated high-frequency acoustic waves. The *Q* was calculated to be 1220 and 240 with and without water contact, respectively. *Q* dropped to approximately ten when a macro size water droplet was dispensed on the top surface of the resonator. The diameter of the water volume was about 100 μm, significantly larger than the acoustic wavelength in water; thus, the thickness of water was practically infinite for the resonator. A lower *Q* means a larger minimum distinguishable frequency shift, *i.e.*, a larger minimum detectable mass. Thus, we have explored ways of improving the *Q* of silicon bulk acoustic resonator in liquids.

### 3.2. Mass Sensitivity Estimation Using Biotin-Streptavidin

**Figure 6.**
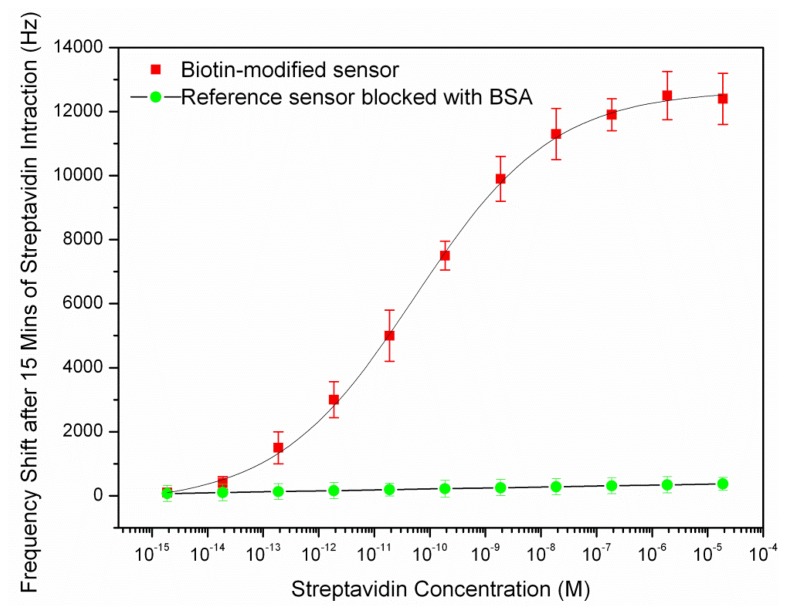
The streptavidin fraction in the solution and corresponding sensor frequency shifts after 15 min of interaction with biotin. The standard deviation in these experiments shown is based on many rounds of the frequency measurements after bonding the streptavidin with coated biotin.

After coating of the resonator surface with biotin-streptavidin, a water droplet spotted in the resonator's center for measurement purpose as described in previous section and the new frequencies were measured. The streptavidin fraction in the solution and corresponding sensor frequency shifts are shown in Figure 6.

As it can be seen in above figure, the curve rises linearly while the available binding sites are being populated and begins to saturate when fewer sites are left available. The dynamic range of the sensor was found between the concentrations of 10^−13^–10^−7^ M. The detection limit of the streptavidin sensor was estimated to be 10–13 M as the frequency shift was not considerably below this concentration. The amount of frequency shift at this concentration corresponded to the measured noise in the previous section (2 KHz). The frequency shift reached to a plateau after putting the biotin-modified chip in the streptavidin solution of 10^−7^ M and the saturation occurred. The reference sensor (the sensor which was blocked with 1 mg/mL BSA) showed a slight frequency shift with increasing analyte concentration with a maximum shift of about 70 Hz at the largest concentration. This shows that the resonator surface had been successfully blocked by BSA pre-treatment.

The resonant frequency of the resonator after saturation is 31.12 MHz that shows a 315 ppm drop compared to the resonant frequency of the sensor before exposing to the streptavidin. The mass sensitivity of the sensor could be calculated from the frequency shift at the saturation and the total deposited mass on the sensor surface. As the streptavidin estimated surface footprint was 4 nm × 5 nm [31], its surface density in the saturation was 5e4 molecules/μm^2^. Given that streptavidin possess molecular weights of 53,000 Da (g/mol), the added mass per unit area was 4.4 fg/μm^2^. This estimated deposited mass was closely matched with the measured mass per unit area of biotin-streptavidin on the FBAR surface, 3.6 fg/μm^2^ [22]. The sensor had an active resonating area of 7.8 × 10^5^ μm^2^, so we further calculated the actual mass increase on the sensor active area due to the biotin-streptavidin interaction was about 3.43 ng. Consequently, the mass sensitivity of the sensor was obtained with replacing the deposited mass per unit area and its corresponding frequency shift in Equation (2). The average mass sensitivity of the sensor in the dynamic range (10^−13^–10^−7^ M), was obtained to be 87.76 μm^2^/ng. The calculated mass sensitivity was almost close with the result obtained from the characterization of the sensor with PEM in water (79.53 μm^2^/ng) in the previous section. The results of the sensor mass sensitivity are summarized as below:

The repeatability of these data was investigated with repeating the above experiment for two similar sensors of the same wafer. The device-to-device variance in the frequency shift was recorded to be 500 Hz, corresponding to 4.3% change in the mean of the frequency shift.

The obtained experimental mass sensitivity of the biosensor in aqueous environment could now be compared with theoretical estimation of Equation (4). The theoretical mass sensitivity for a *L* = 100 μm resonator was obtained to be 96.32 μm^2^/ng, which is about 10% higher than the experimental value. Microresonators operating in the liquid-phase generally suffer from a drastic decrease in their quality factors and mass sensitivity. This decrease in the quality factor increases the frequency noise (which is proportional to *f*/*Q* when operating in an oscillator configuration [32]), thus increasing the limit of detection in biochemical sensing applications.

The limit of detection (LOD) describes the smallest amount of the mass per unit area which produces a measurable output signal considering the existing noise of the resonator. LOD of the biosensor was experimentally estimated in liquid with collecting the resonance frequencies of the resonator in a period of time and calculation of standard deviation. Using the standard deviation and total noise of the resonator (3σ = 2.2 KHz) and Equation (5), the limit of detection of biosensor was estimated to be 23.1 ng/cm^2^.

The resonator configuration case may differ significantly from the intrinsic noise case, since an increase in coating thickness beyond the optimal value may seriously compromise the LOD. This phenomenon has been observed experimentally in [30,33]. As the above mentioned experiments were performed in the normal environment condition, the obtained limit of detection (LOD) includes the unwanted ambient noise (temperature variations) and also measurement equipments' noises (imperfect electrical connections) which are all considered as our measurement limitations. The obtained LOD can be improved by thermally insolating of the test chamber and shielding the electrical connections.

## 4. Conclusions

The capacitive detection of the biotin–streptavidin binding with the mass sensitivity of 87.76 µm^2^/ng in aqueous environment was demonstrated. Contrary to the common method wherein sensor surfaces are coated by a thin gold layer for the immobilization of probe molecules, the immobilization of biotin molecules took place directly on the silicon surface using APTES. A significant frequency change was observed when the interaction takes place on the surface of biotin functionalized resonators as opposed to minor frequency change of unfuctionalized (BSA blocked) sensor. Due to the small size of the resonator they can be mass produced and arrayed for analysis of multiple targets simultaneously. These results encourage the efforts on using Lame mode resonator biosensing and label free analysis of biomolecular interactions.

## Figures and Tables

**Table 1.** Measured resonancefrequency and mass sensitivity of sensorscoated with PEM and Biotin-Streptavidin binding in air and water.

**Added Mass and Test Environment**	**Mass Sensitivity,*S_m_*, (μm^2^/ng)**
PEM coated Resonator, Air	104.23
PEM coated Resonator, Water	79.53
Biotin-Streptavidin, Water	87.76

## Notes

**Authors Contributions** Amir Heidari made substantial contributions in design, microfabrication, sensor characterization, experiments, data analysis, and manuscript preparation. Yong-Jin Yoon made significant contributions in the design and optimization of the sensor, experiment design and manuscript preparation. Woo-Tae Park made contributions in sensor microfabrication troubleshooting and characterization. Pei-Chen Su made contributions in sensor characterization and manuscript preparation. Jianmin Miao provided direction in device design and characterization. Julius Tsai Ming Lin made contributions in designing the process flow and data analysis. Mi Kyoung Park made significant contributions in experiment design, acquisition and analysis of data, and manuscript preparation.
